# RETRACTED: Choi et al. Validation of the CHA2DS2-VA Score (Excluding Female Sex) in Nonvalvular Atrial Fibrillation Patients: A Nationwide Population-Based Study. *J. Clin. Med.* 2022, *11*, 1823

**DOI:** 10.3390/jcm12216770

**Published:** 2023-10-26

**Authors:** Sun Young Choi, Moo Hyun Kim, Hyo Bin Kim, Sa Yul Kang, Kwang Min Lee, Kyung-Yae Hyun, Sung-Cheol Yun

**Affiliations:** 1 Department of Cardiology, Dong-A University Hospital, Busan 49201, Republic of Korea; kmu5041@hanmail.net (S.Y.C.); rlagyqls0112@naver.com (H.B.K.); kangsayul@gmail.com (S.Y.K.); tnt849@hanmail.net (K.M.L.); 2 Department of Biomedical Laboratory Science, Daegu Health College, Daegu 41453, Republic of Korea; 3 Department of Clinical Laboratory Science, Dong-Eui University, Busan 47340, Republic of Korea; kyhyun@deu.ac.kr; 4 Asan Medical Center, Department of Clinical Epidemiology and Biostatistics, University of Ulsan College of Medicine, Seoul 05505, Republic of Korea; ysch97@amc.seoul.kr

The journal retracts the article “Validation of the CHA2DS2-VA Score (Excluding Female Sex) in Nonvalvular Atrial Fibrillation Patients: A Nationwide Population-Based Study” [1]. 

Following publication, methodological and data interpretation concerns were brought to the attention of the Editorial Office.

Adhering to our complaints procedure, an investigation was conducted by the Editorial Office and Editorial Board that confirmed the presence of a number of significant issues with the methods used and the analysis of the data and concluded that the overall findings of this study [1] could not be relied upon. Thus, this article is retracted. 

This retraction was approved by the Editor in Chief of the *Journal of Clinical Medicine*. 

The authors agreed to this retraction. 
